# Robust reconstruction of single-cell RNA-seq data with iterative gene weight updates

**DOI:** 10.1093/bioinformatics/btad253

**Published:** 2023-06-30

**Authors:** Yueqi Sheng, Boaz Barak, Mor Nitzan

**Affiliations:** School of Engineering and Applied Sciences, Harvard University, Boston, MA 02134, United States; School of Engineering and Applied Sciences, Harvard University, Boston, MA 02134, United States; School of Computer Science and Engineering, Racah Institute of Physics, Faculty of Medicine, The Hebrew University of Jerusalem, Jerusalem 9190401, Israel

## Abstract

**Motivation:**

Single-cell RNA-sequencing technologies have greatly enhanced our understanding of heterogeneous cell populations and underlying regulatory processes. However, structural (spatial or temporal) relations between cells are lost during cell dissociation. These relations are crucial for identifying associated biological processes. Many existing tissue-reconstruction algorithms use prior information about subsets of genes that are informative with respect to the structure or process to be reconstructed. When such information is not available, and in the general case when the input genes code for multiple processes, including being susceptible to noise, biological reconstruction is often computationally challenging.

**Results:**

We propose an algorithm that iteratively identifies manifold-informative genes using existing reconstruction algorithms for single-cell RNA-seq data as subroutine. We show that our algorithm improves the quality of tissue reconstruction for diverse synthetic and real scRNA-seq data, including data from the mammalian intestinal epithelium and liver lobules.

**Availability and implementation:**

The code and data for benchmarking are available at github.com/syq2012/iterative_weight_update_for_reconstruction.

## 1 Introduction

Single-cell RNA-sequencing (scRNA-seq) technologies have revolutionized our understanding of heterogeneous cellular populations and the underlying biological processes they encode (Wu et al. 2014; Potter 2018). However, the original spatial context of the cells (and the resulting spatial gene expression patterns) is lost due to the experimental procedure. While multiple computational methods have been developed to reconstruct spatial cellular context (Mages et al. 2023; Nitzan et al. 2019; Satija et al. 2015; Teves and Won 2020; Zeng et al. 2022), this task is challenging, partially because the cells simultaneously encode multiple spatiotemporal processes, and due to technical and biological noise. A common pre-processing step is considering only the highly variable genes as input for such reconstruction algorithms. However, these genes may not all be correlated with the same underlying process and thus may interfere with the reconstruction procedure.

The task of reconstructing cellular structure (or low-dimensional manifold in gene expression space) is tightly related to the task of identifying informative genes with respect to that manifold. On the one hand, a panel of manifold-informative marker genes can yield a metric similar to cell distances on the original structure, thus allowing for the clustering of cells that are close by in the original tissue (Droin et al. 2020). On the other hand, given an embedding of cells, one can recover informative genes with respect to that embedding by viewing individual gene expression as a function of cell location using, for example, regression-type algorithms (Droin et al. 2020). Here, we propose an algorithm that obtains enhanced reconstruction of single-cell data by alternating between these two tasks. By learning a representation of the subset of genes informative for a certain spatiotemporal process (or expressed smoothly along a certain manifold), we show that iterative gene weight update improves the reconstruction quality of existing algorithms, given the weighted gene expression as input, *without relying on prior information*.

##  

We propose an algorithm that iteratively identifies manifold-informative genes, which can be integrated into existing reconstruction algorithms for single-cell data as a *subroutine*. Given single-cell RNA-seq data and a baseline learning algorithm that reconstructs gene expression along a spatial or temporal manifold, our algorithm iteratively updates the weight distribution over genes in the gene expression matrix taken as input to the reconstruction algorithm, to gradually increase the weight over genes related to the underlying reconstructed manifold. This leads to the identification of manifold-informative genes and a better reconstruction of the spatial or temporal manifold encoded by the cells. We demonstrate that such gene weight update improves the quality of reconstruction and identification of manifold-informative genes in several settings, including (i) diverse synthetic single-cell datasets when integrated with an Autoencoder as the baseline algorithm and (ii) over scRNA-seq datasets of mammalian intestine epithelium (Moor et al. 2018) and liver lobules (Droin et al. 2020), when integrated with either an Autoencoder or with novoSpaRc (Nitzan et al. 2019; Moriel et al. 2021) (an optimal-transport-based tissue reconstruction method) as the baseline algorithms.

## 2 Materials and methods

### 2.1 Model description and assumptions

Let $\{x_{1},\ldots ,x_{N}\}\in {\left(R^{n}\right)}^{N}$ be gene expression profiles, where n:=number of genes and N:=number of cells. We assume that each cell, *x*, is represented by its locations on various low-dimensional manifolds $M^{1},\ldots ,M^{k}$, each representing a different biological structure or process. Here, we use location in a generalized way that includes time and additional cell labels belonging to other types of cell structures. However, the true cell locations are generally lost due to the scRNA-seq experimental procedure. For example, the spatial location of cells within the tissue of origin is lost due to cell dissociation. A gene is *correlated* with, or *informative* with respect to a manifold $M_{i}$ if its expression at cell *x* can be written as a smooth function (e.g. a low-degree polynomial) of its location on $M_{i}$.

Definition 1. *Let* $M^{1},\ldots ,M^{k}$*be manifolds where true cell locations are sampled from. For each cell, x, denote the true cell location as*

$$c:=\left[\begin{array}{c} c^{1}\\ \vdots \\ c^{k} \end{array}\right]$$

*where* $c^{i}\in M^{i}$*. Let* $S_{i}$*be the set of genes correlated to* $M^{i}$*s.t.* $\sum_{i}|S^{i}|=n$*. Suppose there exists a set of functions* $f_{1},\ldots ,f_{k}$*s.t.* $f_{i}:M^{i}\to R^{\left|S^{i}\right|}$*and a permutation matrix* $\pi \in {\left\{0,1\right\}}^{n\times n}$*. The gene expression at cell x iswhere* $\left\{y_{x}\right\}$*are i.i.d. samples from a distribution over* $R^{n}$.

Remark 1. *In general, gene expressions are not deterministic functions and we do not assume a subset of genes correlated with the same manifold corresponds to a consecutive subset of coordinates of gene expression vector. In the above definition, we use* π*to include all gene orderings and* $\left\{y_{x}\right\}$*to introduce randomness.*

$$x:=\pi \left(\left[\begin{array}{c} f_{1}(c^{1})\\ \vdots \\ f_{k}(c^{k}) \end{array}\right]\right)+y_{x}$$

Denote $X=[x_{1},\ldots ,x_{N}]\in R^{n\times N}$ as the input matrix where each column is a cell. Given *X*, the goal is to recover $S^{i}$ and the corresponding true cell locations $\left\{c^{i}\right\}$.

Note that some gene *g* may not depend on any cell manifold $M_{i}$. Those genes are not considered informative and we assume gene expression over cells is distributed as independent standard normal random variable.

#### Notation

Denote $\Pi_{n}=\{p\in {\mathbb{R}}_{+}^{n}:||p|{|}_{1}=1\}$ to be the set of distributions over [n]={1,…,n}. Let $u_{n}$ be the uniform distribution over [n]. For S⊂[n], let $\chi_{S}\in \Pi_{n}$ be the uniform distribution over *S* and $p_{S}=|S|\left\langle p_{},\chi_{S}\right\rangle$ be the weight over the set of genes *S*. Let $\Pi_{n}^{c}\subset \Pi_{n}$ be the set of distributions with $||p|{|}_{\infty }\leq c$.

### 2.2 Description of algorithm

To enhance the reconstruction of single-cell data along a certain spatial structure or temporal process, we propose to “boost” the performance of a weak reconstruction algorithm by iteratively shifting the emphasis, or the weight distribution over genes, toward the group of manifold-informative genes using multiplicative weight update (Arora et al. 2012). A pseudo-code description is given in Algorithm 1. Suppose we have a baseline algorithm that can reconstruct cellular location and gene expression along an underlying manifold $M_{i}$ when all the genes used as input to that algorithm correlate with that manifold and suppose we know the subset of genes $S^{i}$. In that case, we can first project the input data *X* onto the subset of genes $S^{i}$ that correlate to $M^{i}$ and use $X^{i}$ as input to the baseline learning algorithm to reconstruct $M^{i}$. Generally, if we do not know $S^{i}$ and the input gene expression matrix contains genes that are not correlated with $M_{i}$, we expect the algorithm’s error to increase. Therefore, our goal is to generate a mask over the input gene expression matrix, which emphasizes, or places a higher probability density, over $S_{i}$. This motivates the following definition.

Definition 2 (Baseline algorithm). *Given X and a distribution p over* {1,…,n}*, a weak algorithm* $A_{k}$*learns a k dimensional projection* $E:R^{N}\to R^{k}$*and* $D:R^{k}\to R^{N}$*s.t. for some *ε>0*, the reconstruction error of* $A_{k}$, $\mathrm{err}(A_{k}(\cdot ))$*, is bounded by some monotone function* $f_{k}$*s.t.* $f_{k}({||\chi_{S}||}_{\bar{S}})=0$

$$X^{i}=\text{diag}(\chi_{S^{i}})X$$

$$\mathrm{err}(A_{k}(\mathrm{diag}(p)X))\in (1\pm \varepsilon )f_{k}(p_{\bar{S}})$$

*where S is a subset of genes recoverable by* $A_{k}$*and* $\bar{S}=[n]/S$.

**Figure 1.**
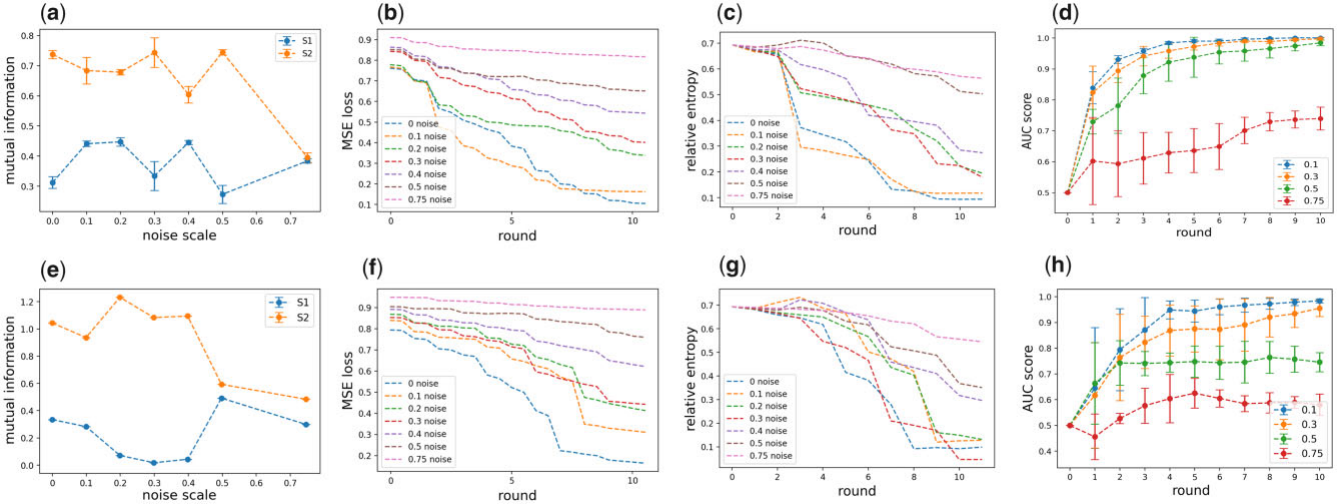
Gene weight updates enhance reconstruction quality over synthetic single-cell data. Experimental results for $D_{1}$ (a–d) and $D_{2}$ (e–h). (a, e) Mutual information between final encoding and gene expressions versus noise scale. Genes are partitioned into two sets $S_{1},S_{2}$ based on the underlying manifold they depend on. We denote $S_{2}$ as a subset of genes that are captured by the AE. For $D_{1}$, $S_{2}$ corresponds to the subset of genes dependent on $\text{Unif}{\left([0,1]\right)}^{2}$, and for $D_{2}$, $S_{2}$ corresponds to the subset of genes whose expression can be expressed as degree 2 polynomials. (b, f) MSE loss versus weight update rounds. (c, g) Relative entropy between algorithm output and ground truth distribution versus weight update round. (d, h) AUC scores for $D_{1}$ and $D_{2}$ versus weight update round over five experiments.

In this work, we use *round* to refer to an iteration in step 3 of Algorithm 1, where we train the baseline algorithm on reweighted data based on the current weights. Maximum number of rounds can be chosen based on application, for example, either terminate the algorithm when the improvement in loss drops below a predetermined threshold (similarly to gradient descent), or when the relative entropy drops below a certain threshold (indicating gene weights concentration on a subset of genes) (Fig. 1c and g).

#### 2.2.1 Baseline algorithms

A baseline reconstruction algorithm, as defined in Definition 2, is a subroutine to our proposed weight update algorithm. The goal of our algorithm is to enhance the performance of the baseline algorithm by focusing on manifold-informative genes for its respective reconstruction.

We test our weight update algorithm over two baseline reconstruction algorithms: feed-forward Autoencoder and novoSpaRc.

*Autoencoder (AE)*. AE takes as input the reweighted data matrix diag(p)X, outputs an encoder $E:R^{D}\to R^{k}$, corresponding to a function that maps input gene expressions to a low-dimensional representation, and a decoder $D:R^{k}\to R^{D}$, corresponding to a function that computes the reconstructed gene expression from the low-dimensional representation. Autoencoders and their variants are widely used for single-cell reconstruction (Eraslan et al. 2019; Amodio et al. 2019; Tran et al. 2021). In this paper, we train a shallow AE with Tanh activation in each round to demonstrate the effectiveness of our weight update algorithm. The largest layer width is set to 64–128, and the code dimension is set to be the minimum cell manifold dimension. The number of epochs is set to 70–100. Our algorithm outputs the subset of genes that can be better reconstructed by AE, which generally corresponds to the subset of genes correlated with a lower dimensional cell manifold or is generated by a simpler class of functions.

*novoSpaRc.* novoSpaRc (Nitzan et al. 2019; Moriel et al. 2021) is a generalized optimal-transport-based method designed to spatially reconstruct single-cell data based on an interpolation between a structural correspondence assumption (corresponding to spatial smoothness of gene expression) and potentially a reference atlas (such as imaging data for a subset of marker genes). novoSpaRc was shown to successfully reconstruct gene expression patterns over diverse tissues, including the intestinal epithelium and liver lobules. We show that our weight update algorithm improves the reconstruction quality of novoSpaRc by making it more robust when input data contains noisy or less informative genes.

#### 2.2.2 Alternative minimization and multiplicative update

Given a baseline algorithm as in Definition 2, we wish to find a distribution *p* that minimizes the reconstruction error of $A_{k}$ while not putting too much weight on any single gene. We do this via the multiplicative weight update algorithm with restricted distributions (Kale 2007). Let l(p,X,D(E(X))) be a loss function that measures the quality of reconstruction. The problem can be rephrased as the following optimization problem: where |S| is the size of the subset of genes correlated with the current cell manifold. That is, we minimize the loss over distributions that put at most $\frac{1}{\varepsilon |S|}$ weight on any particular gene.


$$\min_{p\in \Pi_{n}^{\frac{1}{\varepsilon |S|}}}\underset{E,D}{\min\;\;}l(p,X,D(E(X)))$$

At each round *t*, we compute the optimal encoder and decoder for *X* reweighted by $p^{t-1}$ and update $p^{t}$ by where $D(p,q)=\sum_{i}p_{i}\;\text{log}\;\frac{p_{i}}{q_{i}}$ is the relative entropy. The first term of Equation (1) increases $p^{t}[i]$ for genes with lower reconstruction loss. The second term prevents $p^{t}$ from concentrating on a small subset of genes. Solving for $p^{t}$, where $m^{t}=\nabla l(p^{t},X,D^{t}(E^{t}(X)))$. Finally, we project $p^{t}$ onto $\Pi_{n}^{\frac{1}{\epsilon |S|}}$ as follows: Let *B* be the set of genes with $p^{t}[i]>\frac{1}{\epsilon |S|}$, set $\tilde{p^{t}}[i]=\frac{1}{\epsilon |S|}$ and rescale *p^t^*[*i*] for $i\in \bar{B}$ so that they sum to $1-\frac{\left|B\right|}{\epsilon |S|}$.


(1) $$p^{t}:=\underset{p\in \Pi_{n}}{\arg\;\min}\{\eta \nabla l(p^{t-1},X,D^{t-1}(E^{t-1}(X)){)}^{T}p+D(p,p^{t-1})\}$$

$$p^{t}[i]=\frac{p^{t-1}[i]\;\text{exp}(-\eta m^{t-1}[i]))}{\sum_{j}p^{t-1}[j]\;\text{exp}(-\eta m^{t-1}[j]))}$$

#### 2.2.3 Loss function

Generally, we measure the reconstruction error of $A_{k}$ by a weighted MSE loss: where Y=D(E(X)) is the reconstructed data matrix. The reconstruction error for each gene is and we can rewrite the MSE loss as $\langle m^{t},p^{t}\rangle$.


$$l(p,X,Y)=\frac{1}{N}\sum_{i=1}^{n}p[i]\sum_{j=1}^{N}{\left(X[i,j]-Y[i,j]\right)}^{2}$$

$$m^{t}\left[g\right]=\nabla_{p}\;l\left(p^{t},X,D^{t}\left(E^{t}\left(X\right)\right)\right)[g]=\frac{1}{N}\sum_{x}{\left(x[g]\;-\;D^{t}(E^{t}(x))[g]\right)}^{2}$$

To check that $p^{t}$ is moving in the right direction, one observation is that the marginal of $p^{t}$ over *S* is non-decreasing (Supplementary Information).

For novoSpaRc, we measure reconstruction error by the variance of gene expression over the output cell distribution. Recall that a gene is strongly correlated with the cell distribution if cells that are mapped to similar true cell locations have similar expressions for this gene. Thus, a natural generalization of MSE loss would be the sum of the variance of the genes. In addition, we can also compute the variance of the derivative of the mean gene expressions to enforce the smoothness assumption of true gene expressions. Let $\mu_{1},\ldots \mu_{h}:[N]\to [0,1]$ be the cell distribution computed by novoSpaRc where *h* is the number of cell locations.


$$\begin{array}{c} l(p,X,\mu_{1},\ldots ,\mu_{h})=\sum_{i=1}^{n}p[i][\left(1-\lambda \right)\sum_{j=1}^{h}V{\text{ar}}_{\mu_{j}}(X[i])\\ +\lambda {\text{Var}}_{j}(E_{\mu_{j}}[X[i]]-E_{\mu_{j-1}}(X[i]))] \end{array}$$

where ${\text{Var}}_{j}$ is the empirical variance over true cell locations and λ in [0, 1].

Algorithm 1. Alternate minimization

**Require:** $X\in R^{n\times N}$ , ϵ

1: $p^{0}⇐u_{n}\;t⇐0$

2: **for**t=0,1,…**do**

3:    $\left(E^{t},D^{t})=A_{k}(\text{diag}(p^{t})X\right)$

4:    $m^{t}=\nabla_{p}l(p^{t},X,D^{t}(E^{t}(X)))$

5:    $p^{t+1}[g]\propto \;\text{exp}(-\eta m^{t}[g])p^{t}[g]$

6:    Project $p^{t+1}[g]$ onto $\Pi_{n}^{\frac{1}{\epsilon |S|}}$

#### 2.2.4 Evaluation

On Synthetic datasets, we evaluate the performance of the algorithm in the following way:

1. *The encoder captures the true cell location for the corresponding cell manifold*: we compute the mean and standard mutual information between the encoding of cells and gene expressions for each subset of genes.
2. The algorithm achieves better reconstruction of gene expression after each update of p and D, E: reflected by a decrease in the MSE loss after each update.
3. *The algorithm recovers the correct subset of informative genes*: reflected by a decrease in the relative entropy between the ground truth distribution and $p^{t}$, $D(\chi_{S},p^{t})$.
4. *Round* $p^{t}$*to recover the correct subset of genes*: AUC score increases. By giving all genes s.t. $p^{T}[i]≳p^{0}[i]$ the same label, we show that the true positive rate remains ≥90% while the false-positive rate decreases after  10 iterations.

For the real scRNA-seq datasets, we compute the Pearson’s correlation coefficients between known and reconstructed mean gene expressions. We first run the baseline algorithm on pre-weighted (original) data (output of round 0) and show that the Pearson’s correlation coefficients improve after ∼10 rounds of weight update.

### 2.3 scRNA-seq simulations

Table 2 is a summary of the simple synthetic single-cell datasets when genes correlate with a single-cell manifold. We first generated $2\times 10^{4}$ synthetic cell locations. For each cell manifold, we simulated single-cell RNA-seq data by generating random low-degree polynomials as functions for gene expression. Each gene is normalized to have a mean of 0 and a variance of 1. To obtain a mixture of datasets, we stack two datasets containing genes correlated with a single-cell manifold so that each cell contains genes correlated with two manifolds. Table 3 contains a summary of mixtures of datasets.

**Table 2.** Synthetic datasets.

Cell distribution	Function type
Unif([0,1]) , $\text{Unif}{\left([0,1]\right)}^{2}$	Polynomials with degree ≤3
N(0,1) , $N(0,I_{2})$	Polynomials with degree ≤3

**Table 3.** Mixture datasets.

Type of mixture	Cell location	Gene expression
Different cell locations distribution ($D_{1}$)	$\text{Unif}{\left([0,1]\right)}^{2}$ , $N(0,I_{2})$	Degree 3 polynomials
Different degree for gene expression ($D_{2}$)	N(0,1)	Degree 2 and 3 polynomials
Different cell manifolds dimension ($D_{3}$)	$\text{Unif}{\left([0,1]\right)}^{2}$ and Unif([0,1])	Degree 3 polynomials
Different cell manifold dimension and gene expression degree ($D_{4}$)	N(0,1) and $N(0,I_{2})$	Degree 2 and 1 polynomials

When adding Gaussian noise to the data, we say it has *noise scale* β if the input is of the form where *X* is the noise-less data as described above and $\bar{N}$ is the normalized noisy data where each entry N[i,j]∼N(0,1). Each gene expression in $X_{\beta }$ has a mean of 0 and variance of 1.


$$X_{\beta }=\sqrt{1-\beta }X+\sqrt{\beta }\bar{N}$$

### 2.4 scRNA-seq datasets

We analyze two publicly available single-cell RNA-seq datasets. In both datasets, the reconstructed low-dimensional manifolds correspond to one-dimensional spatial axes within the tissues of origin (crypt-to-villus axis in the intestinal epithelium, portal-to-central vein in liver lobules). Generally, zonated genes are genes whose expression can be expressed as a function of the manifold corresponding to the spatial axes.

The first dataset contains cells sampled along the crypt-to-villus axis in the mammalian intestinal epithelium (Moor et al. 2018). In the original study, cell locations are partitioned into seven spatial layers. Here, we denote zonated genes as highly expressed zonated genes with Q value <0.05 as given by Moor et al. (2018).

The second scRNA-seq dataset contains hepatocytes sampled from mammalian liver lobules (Droin et al. 2020). In the original study, genes were classified as being correlated to either spatial zonation across the liver lobule axis (space), or to the circadian rhythm (time), or to their combination. The two cell manifolds, space and time, are denoted as c=(s,t)∈{0,…,7}×{0,…,3}, respectively. Specifically, genes in (Droin et al. 2020) were classified into the following disjoint groups:

- Space-dependent genes (Z): $z_{g}(s)=as^{2}+bs+c$
- Time-dependent genes (R): $r_{g}(t)=a\;\text{sin}(\omega t+b)$
- Space + time genes (Z + R): $f_{g}(s,t)=az_{g}(s)+br_{g}(t)$
- Space × time genes (Z × R): $f_{g}(s,t)=az_{g}(s)r_{g}(t)$
- Unlabeled genes: noisy genes or low variance genes across space and time.

where *a*, *b*, *c* are constants that are different for each gene. Notice that there is no need to explicitly model the gene expression for the use of the algorithm we propose for iterative weight updates: for the liver data it is used only for evaluation; for the intestine data it is not used at all.

In both cases, gene expression was pre-processed and normalized, as commonly done for scRNA-seq reconstruction algorithms (Moor et al. 2018; Nitzan et al. 2019; Droin et al. 2020), by log-transform, and z-score normalization.

## 3 Results

### 3.1 Reconstruction of synthetic single-cell datasets

We first evaluate the performance of our algorithm on various mixtures of synthetic single-cell datasets to showcase the identification of manifold-informative genes and an encoding of the corresponding cell location when signal-to-noise ratio <1. The synthetic datasets (Table 3) are mixtures of basic single-cell datasets (Table 2) that differ in cell manifolds dimension, distribution, and degree of gene expression, with noise scale β∈[0,0.75]. Employing iterative gene weight updates over single-cell reconstruction using an Autoencoder output improves both relative entropy and accuracy of reconstruction (Fig. 1, Table 1 and Supplementary Fig. S1). Specifically, iterations of gene weight updates lead to the gradual decrease in relative entropy between the output distribution and ground truth, decrease in gene expression reconstruction error, increase in AUC score for gene expression reconstruction, and decrease in false-positive rate (and consistently high, >93%, true positive rate) for manifold-informative genes (where in each case of the synthetic mixtures, the correct subset of manifold-informative genes could be obtained by thresholding their weight by $\frac{1-\delta }{n}$ for some small δ=0.2) (Fig. 1 andSupplementary Figs S1–S5). The quality of reconstruction and gene partition gradually decreases with the increasing noise level, as expected (Fig. 1b and f and Supplementary Figs S1b and f and S2b–S5b).

### 3.2 Reconstruction of scRNA-seq intestinal epithelium data

**Figure 2.**
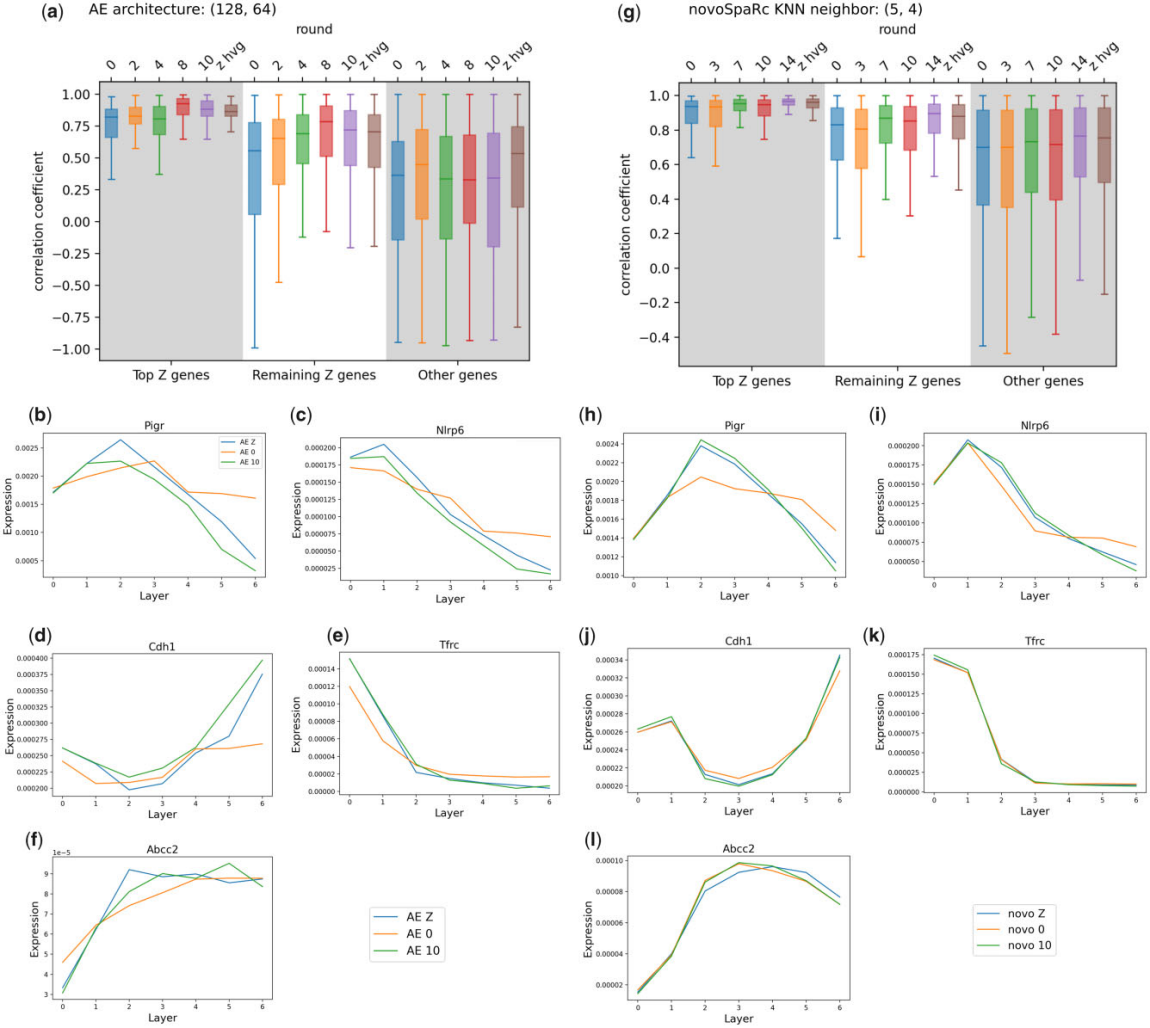
Iterative gene weight updates enhance spatial reconstruction of the intestinal epithelium. (a, g) Quality of reconstruction measured by correlation coefficients following weight updates when using an Autoencoder (a) or novoSpaRc (g) as a baseline algorithm. *z hvg* corresponds to the output when trained on the subset of zonated, highly variable genes. (b–l) Mean gene expressions for sample genes from each of the five clusters of zonated genes in (Moor et al. 2018), reconstructed by novoSpaRc (h–l) and AE (b–f) after 0 and 10 rounds of update (novo or AE 0 or 10, respectively), compared to when only trained on zonated highly variable genes (novo or AE Z).

Here, we show that coupling reconstruction to iterative gene weight update as a subroutine can improve the quality of tissue reconstruction based on scRNA-seq data. We first focus on scRNA-seq data from the mammalian intestinal epithelium (Moor et al. 2018). To capture spatial gene expression along the crypt-to-villus axis, we generated a one-dimensional embedding of the cells, based on the top 1000 highly variable genes (approximately 50% of which are labeled as zonated by Moor et al. 2018), using both the baseline Autoencoder and novoSpaRc. In both cases, iterative weight updates gradually improve the quality of tissue reconstruction (Fig. 2). Specifically, when tracking the correlation between the reconstructed and measured gene expression values, we find that the quality of reconstruction of spatially zonated genes improves with each iteration of the gene weight update (Table 1). Following 10 rounds of gene weight update, the reconstruction quality matches that of training only on zonated, highly variable genes (Fig. 2).

Furthermore, our algorithm gradually shifts the distribution of weights during training, as shown by the decrease in relative entropy between the current weight distribution and uniform distribution over zonated genes with weight update rounds (Supplementary Fig. S8). While novoSpaRc’s quality of reconstruction is higher than that of the baseline Autoencoder, the qualitative effects described in this section are shared by both algorithms (Fig. 2).

### 3.3 Reconstruction of scRNA-seq liver data

We next focus on a more challenging scenario, when genes change along two structures or processes, encoded by multiple cellular manifolds, simultaneously. This is the case for hepatocytes within liver lobules, whose expression changes across both physical space (along the lobule axis) and circadian time, as reflected in a recently collected scRNA-seq dataset (Droin et al. 2020).

**Figure 3.**
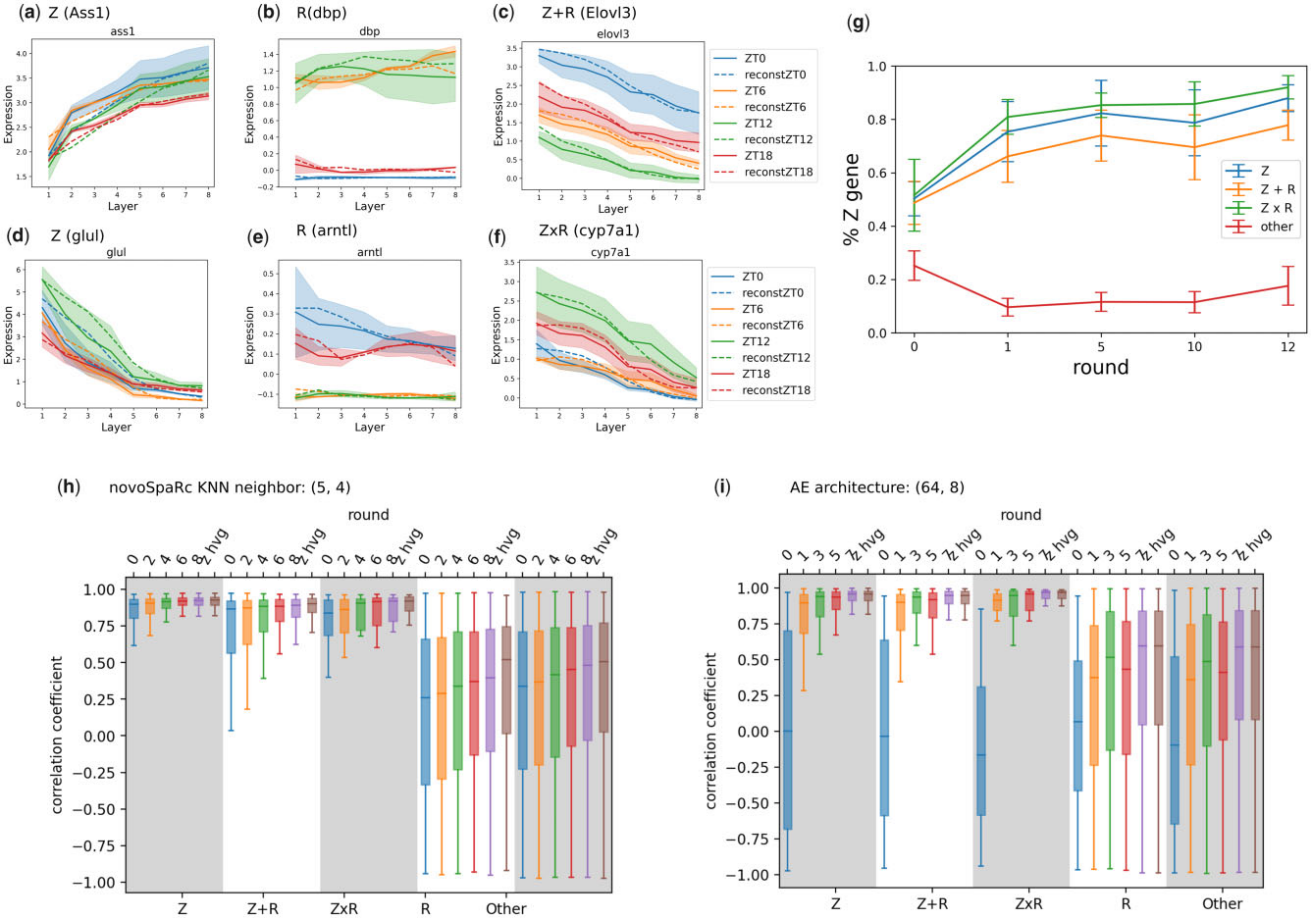
Iterative gene weight updates enhance spatial reconstruction of liver lobules. (a–f) Example outputs of the recovered mean gene expression based on the iterative weight update algorithm, compared to the true mean gene expression. ZT are ground truth mean gene expressions and reconst ZT are reconstructed mean gene expressions after 10 rounds of weight update using AE as the baseline algorithm. (g) Percentage of zonated genes labeled by the weight update algorithm for *Z*, Z+R and Z×R genes as well as non-zonated genes. Error bars are computed over five runs of training. (i, h) The improvement in reconstruction quality was measured by correlation coefficients after 10 rounds of weight updates for AE (i) and novoSpaRc (h). *z hvg* corresponds to the output when trained on the subset of zonated highly variable genes.

We construct a one-dimensional embedding of the cells using both an Autoencoder and novoSpaRc as baseline algorithms. We use the top highly variable genes (200 for AE and 500 for novoSpaRc) out of 15,000 genes for training, approximately 50% of which are zonated (Z, Z + R, or Z × R) genes.  Applying our algorithm for iterative weight updates gradually enhances the quality of reconstruction of zonated genes (Z, Z + R, Z × R) in the de novo setting (without using a reference atlas) (Table 1), and achieves comparable results to those obtained by using a reference atlas as prior knowledge (Fig. 3). This occurs due to the increased weight density over spatially informative genes achieved by the iterative weight update.

Furthermore, we are able to recover the correct labels for most zonated genes (Z, Z + R, Z × R) using the final cell embedding output by the algorithm, as described below. Similarly to Droin et al. (2020), we recover gene labels from the cell embedding by using a linear regression model to approximate (reconstructed) mean gene expression by low-degree polynomials of *s* and *t*. In (Droin et al. 2020), a mixed regression model was used to approximate measured mean gene expression, however, since we focus here on spatially informative genes whose expression changes monotonically with space, the zonated part of gene expression could be approximated by a monotone function captured by linear regression. Thus, we fit each mean gene expression to a linear function and label genes that can be approximated well (*R* score >0.1) and are informative with respect to the spatial manifold (slope >0.001). Our algorithm achieves 93.7% true positive rate on purely zonated genes and >80% of true positive rate on mixed zonated genes (Fig. 3g).

## 4 Discussion

Our algorithm for iterative gene weight update improves the accuracy of single-cell RNA-seq reconstruction. Our algorithm can reconstruct gene expression, including the case when multiple cell manifolds are simultaneously encoded, without depending on prior information such as manifold-informative marker genes. We showcase these results over two baseline reconstruction algorithms, an Autoencoder and novoSpaRc, and over synthetic single-cell data as well as two scRNA-seq datasets of mammalian intestinal epithelium and liver lobules. Moreover, our algorithm can be incorporated into a general baseline reconstruction algorithm, treating it as a blackbox. Therefore, while in this manuscript we focus on enhancing spatial reconstruction algorithms that are based on scRNA-seq data alone, our algorithm for iterative weight update can also be applied to the reconstruction of underlying structures in the data corresponding to non-spatial processes such as temporal trajectories (see benchmarking of trajectory inference methods in Saelens et al. 2019), as well as algorithms that combine multiple types of experimental data, such as scRNA-seq and spatial transcriptomics data for spatial reconstruction. While our algorithm requires the selection of an appropriate loss function for the chosen baseline algorithm that effectively measures the reconstruction quality of each gene, it can generally be informed by the objective and characteristics of the baseline algorithm.

## Supplementary Material

btad253_Supplementary_Data

## Data Availability

The scRNA-seq datasets used for this study were acquired from the Gene Expression Omnibus (GEO) database with the following accession numbers: liver (GSE145197), intestine (GSE109413). Reconstructed mean gene expression for the liver data was downloaded from (https://github.com/naef-lab/Circadian-zonation). Zonated gene labels for the intestine were downloaded from (https://zenodo.org/record/1320734). The data and code for generating synthetic single-cell datasets are included in github.com/syq2012/iterative_weight_update_for_reconstruction. Summary of results. Summary of reconstruction results on synthetic and scRNA-seq datasets for the baseline algorithms (Autoencoder and novoSpaRc) without and with the integration of the weight update algorithm as a subroutine. For real scRNA-seq datasets, we record the mean and variance of correlation coefficients between reconstructed and ground truth zonated gene expressions. For Synthetic datasets, we measure the MSE loss on different noise levels. Higher correlation coefficients and lower MSE loss indicate better gene expression reconstruction. In both cases, the integration of the weight update algorithm enhances the quality of reconstruction.
