# Supplementary material for: Robust reconstruction of single-cell RNA-seq data with iterative gene weight updates
Source: Bioinformatics. 2023 Jun 30;39(Suppl 1):i423–30. doi: 10.1093/bioinformatics/btad253 (PMC10311330; doi:10.1093/bioinformatics/btad253)
Supplement: btad253_Supplementary_Data [file btad253_supplementary_data.pdf]

1 Supplementary Figures

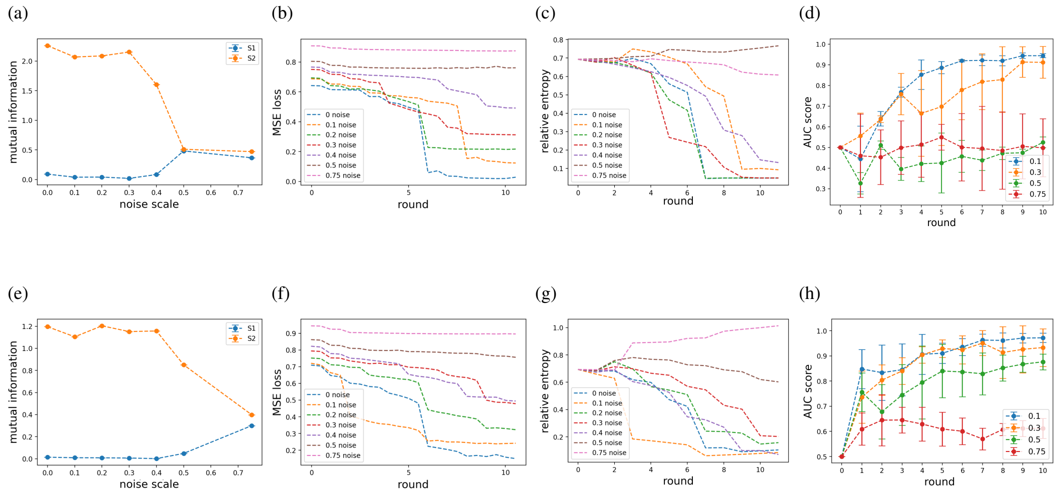

Fig. S1: **Gene weight updates enhances reconstruction quality over synthetic single-cell data.** Experimental results for D_3 (First row) and D_4 (second row). S1a, S1e) mutual information between final encoding and gene expressions vs noise scale, where S_2 are a subset of genes that are captured by the AE. For D_3 and D_4, this is the subset of genes correlated with $\text{Unif}([0,1])$ and $\text{Unif}([0,1])^2$ respectively. S1b , S1f) MSE loss vs weight update rounds. S1g, S1g) relative entropy between algorithm output and ground truth distribution vs weight update round. S1d, S1h) AUC scores for D_3 and D_4 vs weight update round over 5 experiments.

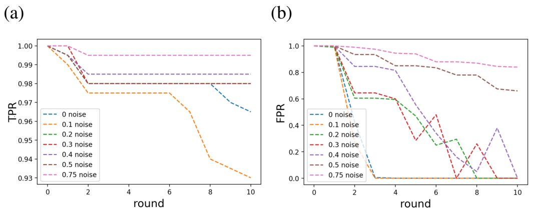

Fig. S2: True positive and false positive rate for D_1

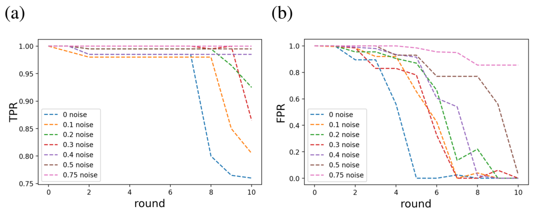

Fig. S3: True positive and false positive rate for D_2

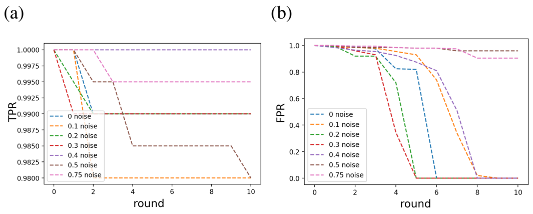

Fig. S4: True positive and false positive rate for D_3

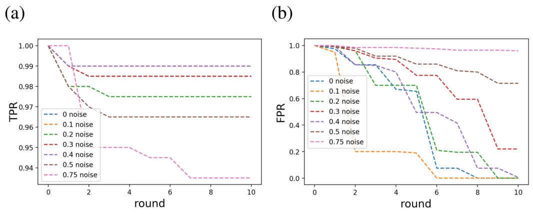

Fig. S5: True positive and false positive rate for D_4

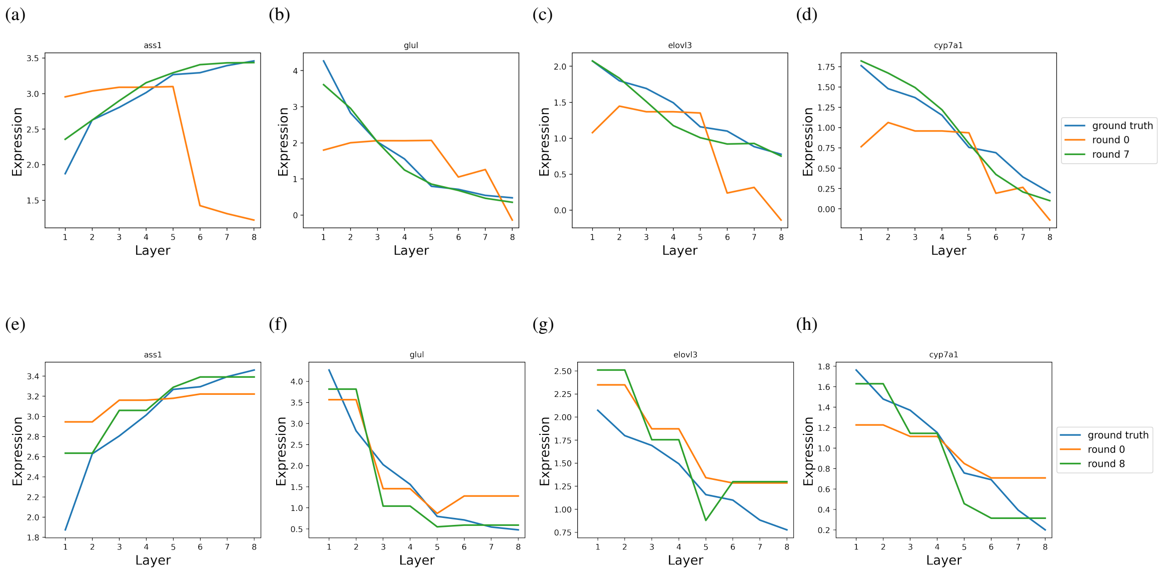

Fig. S6: **Mean gene expression of zonated genes in the liver single-cell data (Droin et al., 2020)** when trained on highly variable genes (at round 0) and following 7 rounds of weight updates, compared to the ground truth mean gene expression. S6a - S6d are plots generated when using Autoencoder as the baseline algorithm; S6e - S6h are plots generated when using novoSpaRc as the baseline algorithm.

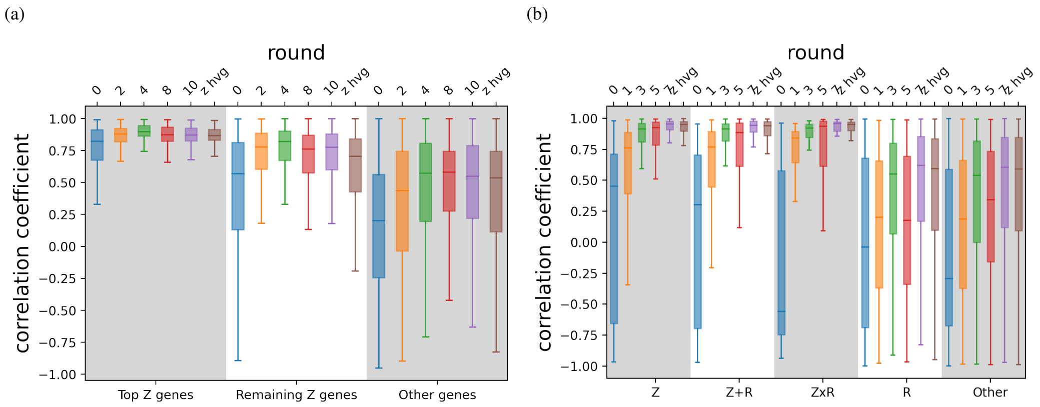

Fig. S7: **Reconstruction quality after 10 rounds of weight updates for Autoencoder trained with 200 epochs.** S7a is the plot of correlation coefficients for intestine dataset; S7b is the plot of correlation coefficients for the liver dataset.

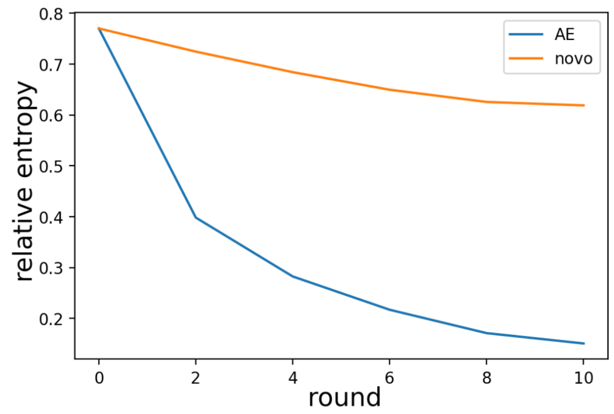

Fig. S8: Relative entropy between current weight and uniform distribution over all highly zonated genes, as a function of weight update rounds, for the intestinal scRNA-seq data

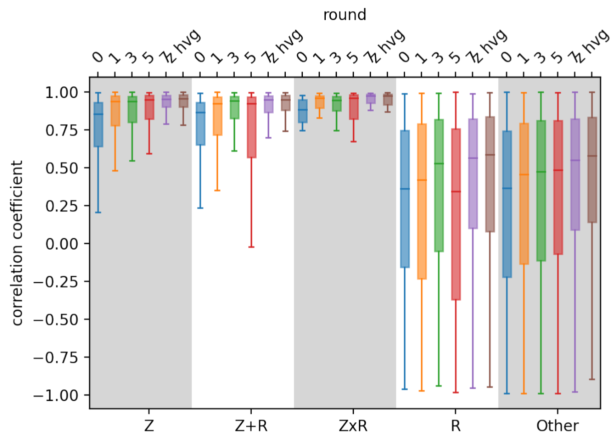

Fig. S9: Reconstruction quality, measured by correlation coefficient, on the liver dataset after 10 rounds of weight updates for Autoencoder trained on highly variable genes with $\geq 90\%$ non zero cell expression.

2 Proof for Lemma 1

Lemma 1. *For the baselines algorithm in Alg 1 with* $err(A_k(\cdot)) := l(\cdot, E, D)$ *and* $\eta_t \leq 2\,\|\mathrm{m}^{\mathrm{t}}\|_\infty$, $\partial_t |S| \langle \chi_S, p^t \rangle > 0$. *Moreover, the algorithm reaches* $p^t_{\bar{S}} < \delta$ *in* $O(\frac{1-\epsilon}{\delta})$ *steps.*

Proof. For $t \in [a, a+1]$, $m^t = m^a$. By assumption, $\langle p^t, m^t \rangle \in (1 \pm \epsilon) f(p^t_{\bar{S}})$. To bound $\langle p^t, m^t \rangle_{\bar{S}}$, we look at the marginal distribution of p^t on $\bar{S}$, denote as $\frac{1}{p^t_{\bar{S}}} p^t|_{\bar{S}}$. Since $m^t|_{\bar{S}}$ can not do better than the optimal solution of $A_k(X, \frac{1}{p^t_{\bar{S}}} p^t|_{\bar{S}})$,

$$\langle p^t, m^t \rangle_{\bar{S}} \geq p^t_{\bar{S}} (1-\epsilon) f(1)$$

Using $\partial_t p^t[i] = -p^t[i](m^t[i] - \langle p^t, m^t \rangle)$ to compute the gradient at $t = a$.

$$\begin{aligned}
\partial_t \langle \chi_S, p^a \rangle &= -\langle p^a, m^a \rangle_S + p^t_S \langle p^a, m^a \rangle \\
&= (1 - p^a_{\bar{S}}) \langle p^a, m^a \rangle - \langle p^a, m^a \rangle_S \\
&= \langle p^a, m^a \rangle_{\bar{S}} - p^a_{\bar{S}} \langle p^a, m^a \rangle \\
&\geq p^a_{\bar{S}} (1-\epsilon) \left(f(1) - f(p^a_{\bar{S}}) \right)
\end{aligned}$$

For $p^t_{\bar{S}} \in [0,1]$, since f is monotone $\partial_t |S| \langle \chi_S, p^a \rangle \geq 0$. To choose the step size,

$$\begin{aligned}
\partial_{t^2} \langle p^t, \chi_S \rangle &= |S| \sum_{i \in S} \partial_t \left(p^t[i](\langle p^t, m^a \rangle - m^a[i]) \right) \\
&= \sum_{i \in S} \partial_t p^t[i] \left(\langle p^t, m^a \rangle - m^a[i] \right) + p^t[i](\langle \partial_t p^t, m^a \rangle) \\
&= \partial_t \langle p^t, \chi_S \rangle \langle p^t, m^a \rangle - \langle \partial_t p^t, m^a \rangle_S + p^t_S \partial_t \langle p^t, m^a \rangle
\end{aligned}$$

Since $\partial_t \langle p^t, m^a \rangle < 0$, $|\partial_{t^2} \langle p^t, \chi_S \rangle| \leq (2|\partial_t \langle p^t, \chi_S \rangle|)\,\|\mathrm{m}^{\mathrm{a}}\|_\infty$. Let $\eta \leq \frac{1}{4\,\|\mathrm{m}^{\mathrm{a}}\|_\infty}$, for $t \in [a, a+\eta)$

$$\begin{aligned}
\partial_t \langle p^t, \chi_S \rangle &= \partial_t \langle p^a, \chi_S \rangle + \int_0^t \partial_{t^2} \langle p^s, \chi_S \rangle \, ds \\
&\geq \partial_t \langle p^a, \chi_S \rangle - 2\,\|\mathrm{m}^{\mathrm{a}}\|_{\int_0^t} \infty \partial_t \langle p^s, \chi_S \rangle \, ds \\
&\geq \partial_t \langle p^a, \chi_S \rangle - t2\,\|\mathrm{m}^{\mathrm{a}}\|_\infty \, \partial_t \langle p^a, \chi_S \rangle \geq 0
\end{aligned}$$

Let T be the first time $p^T_{\bar{S}} < \delta$, the total change of p^t_S for $p^t_{\bar{S}} > \delta$ is

$$\begin{aligned}
1 > p^T_S - p^0_S &= \sum_{i=1}^{T} p^i_S - p^{i-1}_S \\
&\geq \frac{\eta}{2} \sum_i \partial_t \langle p^i, \chi_S \rangle \\
&\geq T \frac{\delta}{2} (1-\epsilon)(f(0) - f(\frac{|\bar{S}|}{D}))
\end{aligned}$$

$T < \frac{2(1-\epsilon)(f(0) - f(\frac{|\bar{S}|}{D}))}{\delta}$
